# Difficulty in the Clinical Diagnosis of Tularemia: Highlighting the Importance of a Physical Exam

**DOI:** 10.1155/2018/9682815

**Published:** 2018-03-01

**Authors:** Rupin Kumar, Mohamed Mansour, Jacqueline Brunetto, Renuka Verma, Margaret Fisher, Jonathan Teitelbaum

**Affiliations:** Department of Pediatrics, The Unterberg Children's Hospital at Monmouth Medical Center, Long Branch, NJ, USA

## Abstract

We report an 18-month-old male who presented with fever and nonspecific symptoms. He was evaluated for multiple differential diagnoses including Kawasaki disease and JIA and received treatment for them. After he was readmitted, tularemia was considered based on the physical exam finding of an ulcer on the scalp and enlarged lymph nodes. Tularemia titers were positive, and the patient was given the appropriate antibiotic and was discharged home. Follow-up of the patient showed complete resolution of symptoms. This is a case that demonstrates the importance of physical exam in identifying rare diseases presenting with common signs and symptoms.

## 1. Introduction

*Francisella tularensis* is the causative agent of the bacterial zoonotic disease tularemia, which is mostly endemic to the northern hemisphere [1]. The incidence of cases reported in the United States nearly doubled, from 180 in 2014 to 314 in 2015. New Jersey, however, continues to have a low incidence rate, with just 1 case reported in 2015 [2, 3]. Depending on the mode of inoculation, the presentation may vary, from localized papule formation and tender lymphadenitis to flu-like symptoms, exudative pharyngitis and tonsillitis [4, 5]. Such nonspecific presenting symptoms may overlap with symptoms of other diseases, including Kawasaki disease as in our case [6]. The appearance of a black eschar over the tender ulcerated lesion at the site of inoculation, which is a more specific diagnostic finding, may take 7–10 days to appear [7]. Hence, initial symptoms can be deceptive and require a high index of suspicion to make the correct diagnosis.

Here, we describe the case of an 18-month-old boy, presumptively treated for incomplete Kawasaki disease before being correctly diagnosed with tularemia.

## 2. Case Presentation

An 18-month-old male with a 3-day history of fever, cough, rhinorrhea, and a nonpruritic, diffuse confluent rash on the extensor surface of both legs presented to our Emergency Department (ED). Viral PCR panel was positive for parainfluenza virus, and he was discharged the same day. The following day, he developed swelling of both hands and feet with tender right-sided posterior cervical lymphadenopathy, along with persistent fever. Laboratory testing revealed leukocytosis and elevated inflammatory markers. Based on concerns of an incomplete presentation of Kawasaki disease, he was admitted to the hospital.

**Figure 1:**
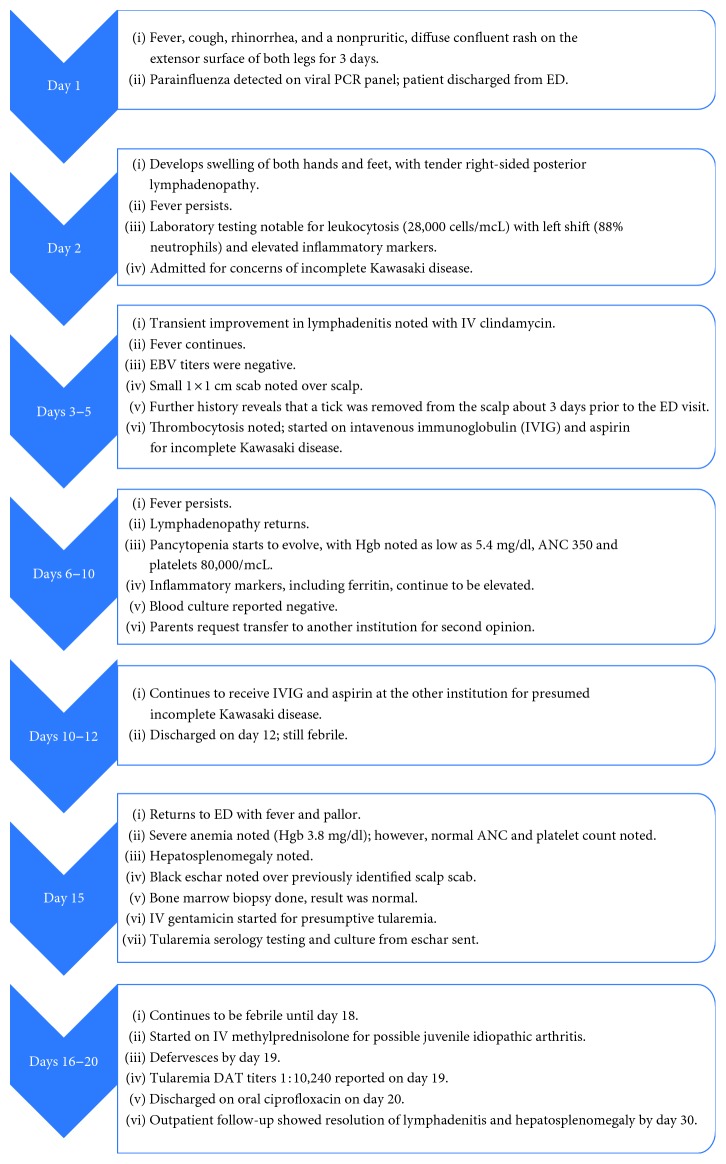
Timeline of symptoms and management.

His hospital course and management are summarized in Figure 1.

**Figure 2:**
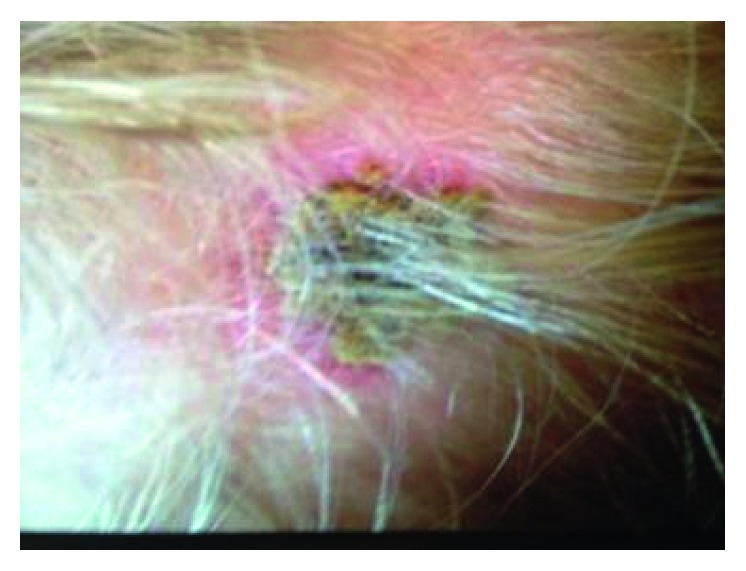
Scab on the skull.

The patient was initially started on IVIG and high-dose aspirin as well as IV clindamycin as empiric therapy for lymphadenitis. Careful physical examination revealed a small 1 × 1 cm scab on the scalp (Figure 2). Upon further questioning, parents revealed that they had removed a tick from the area about 3 days prior to the ED visit. Since the onset of current symptoms was prior to the tick bite, it was considered unlikely at this time that this was causative. Testing for Lyme disease was not done as the patient did not have signs suggestive of Lyme disease such as erythema migrans, musculoskeletal complications, or CNS manifestations.

He returned to our ED three days after discharge, with severe anemia, and was still febrile. Physical examination revealed a moderately enlarged liver and spleen and a black eschar formed over the previously identified scab on the scalp. At this point, the possibility of hemophagocytic lymphohistiocytosis (HLH) syndrome was entertained; however, the triglyceride level was normal, while ferritin was moderately elevated, which is expected in any acute inflammatory process. He was transfused packed RBCs and underwent bone marrow biopsy, which was unremarkable. Repeat blood culture and culture from eschar were reported negative. Given the history of tick bite, the serum sample for tularemia antibody serology was sent, and the patient was started empirically on IV gentamicin. Other diagnostic possibilities considered were autoimmune hemolytic anemia, juvenile idiopathic arthritis, and IVIG-induced hemolysis. Hemolysis was ruled out based on a normal reticulocyte count and negative direct and indirect Coombs tests. The patient received a dose of methylprednisolone for presumed juvenile idiopathic arthritis. Fever resolved by day 19. The tularemia direct agglutination test was reported positive on day 19, with a titer of 1 : 10,240 (*F. tularensis* AB, direct agglutination test performed at Focus Diagnostics, Inc., San Juan Capistrano, California, USA). He was diagnosed with the glandular form of tularemia and discharged on oral ciprofloxacin on day 20.

## 3. Discussion

Tularemia can spread through various modes of transmission: direct contact with infected animals, handling of infectious animal tissues or fluids [8], ingestion of contaminated food, water, or soil, and exposure to a laboratory setting. The causative agent *Francisella tularensis* has been classified as a Category A bioterrorism agent by the CDC. Person-to-person transmission has not been documented [9]. Most cases in the US have been associated with bites from infected arthropods, commonly *Amblyomma americanum* (lone star tick), *Dermacentor variabilis* (dog tick), and *Dermacentor andersoni* (*wood tick*) [10, 11].

Depending on the mode of entry, the disease can manifest itself in various ways with ulceroglandular disease accounting for 45–85% of all cases. Glandular, pneumonic, oculoglandular, and oropharyngeal diseases account for the remaining presentations [12]. Once a tick has inoculated the subject, the organisms proliferate locally and cause a papule to develop within 3–5 days. This inflammatory nidus later becomes necrotic, forming an ulcer, which is progressively replaced by a black eschar. Organisms can then spread from this site to regional lymph nodes causing tender lymphadenitis [7, 13].

In tick-borne tularemia, it has been reported that 50% of patients had the ulcer located on a lower extremity or the perineal area and 30% on the trunk, while very few cases of scalp ulceration were found [14]. However, not all cases of scalp eschar and tender lymphadenitis represent a tularemia infection. In 1997, Lakos reported 27 cases of tick-transmitted infection with occipital eschar and painful lymphadenopathy in the region of the tick bite, which he named “tick-borne lymphadenopathy or TIBOLA” [15]. Later in 2010, Angelakis et al. discovered a case of TIBOLA caused by *Bartonella henselae* and renamed this syndrome “scalp eschar and neck lymphadenopathy after tick bite (SENLAT)” [16]. Since then, several organisms causing SENLAT have been described, namely, *Rickettsia slovaca*, *Rickettsia raoultii*, and *Francisella tularensis* [17]. In about 25% cases, the causative organism could not be identified [18].

Our patient initially received treatment for incomplete Kawasaki disease given his clinical presentation with fever, pharyngitis, tender lymphadenopathy, and a nonspecific rash. Careful physical examination revealed evolution of the scalp eschar, which prompted evaluation for tularemia. The source of infection was thought to be a tick bite on the scalp. A retrospective review of 121 cases published in 2012 detailed that 25% of infected subjects were anemic and 11% had thrombocytopenia [19]. This fact, along with possible transient bone marrow suppression by the concurrent parainfluenza infection, can explain the initial pancytopenia in our patient. In general, tularemia is usually not associated with dramatic changes in white blood cell count, which may be normal or elevated, and differential count typically shows a relative increase of mononuclear cells [20]. Hepatosplenomegaly has also been described, although later in the course of the disease [21]. The possibility of other viral infections such as EBV, CMV, HIV, and parvovirus as well as cat-scratch disease was also considered, although negative serology was reported for all.

Our patient's presenting symptoms were initially attributed to a viral cause; however, persistent fever with bilateral lymphadenopathy and elevated inflammatory markers steered the diagnosis towards incomplete Kawasaki disease. Since the onset of symptoms was prior to the history of tick bite, tick-related etiology was not entertained in the beginning. As pancytopenia and hepatosplenomegaly evolved, our differential diagnoses encompassed viral bone marrow suppression, antibody-mediated hemolysis, juvenile idiopathic arthritis, and hemophagocytic lymphohistiocytosis (HLH syndrome). Careful physical examination with discovery of the formed eschar over the site of tick bite was what clinched the diagnosis.

A presumptive diagnosis of tularemia can be made if a single serum antibody titer is at least 1 : 160 by tube agglutination (TA) or at least 1 : 128 by microagglutination (MA); however, this can also represent past infection. Diagnosis is confirmed if there is a fourfold or higher increase in the titer between acute and convalescent serology with one specimen having a minimum titer of 1 : 160 by TA or 1 : 128 by MA [1]. However, it is important to remember that serology may remain negative for the first 7 to 14 days of infection [22].

Antibiotic therapy should be initiated as soon as tularemia is suspected, rather than awaiting results of serologic testing. Gentamicin is the drug of choice for the treatment of tularemia in children. Ciprofloxacin is an alternative for mild disease [1].

Delayed diagnosis of tularemia can prove to be fatal. If left untreated, complications ranging from suppurative adenitis to hepatic and renal failure and fulminant septicemia have been described in literature [3].

## 4. Conclusion

Tularemia is a tick-borne illness that can present with nonspecific symptoms of fever and lymphadenitis. The initial presentation can be confused with other entities such as Kawasaki disease. The presence of a black eschar at the site of a tick bite should alert the clinician to the possibility of tularemia. This case exemplifies the importance of a careful physical exam and, more so, highlights the requirement of close follow-up and serial exams to track evolution of new physical signs. A high index of suspicion is especially required for rarer entities presenting with rather common symptoms.
